# Michigan as a Recruiting Field

**Published:** 1900-07

**Authors:** 


### MICHIGAN AS A RECRUITING FIELD.

Upon another page of this issue we publish a series of comments and doings in Michigan. They will not fill the minds of those who so strongly advocated the "City of Straits" as the meeting place for 1900 with the same degrees of pleasure and delight that perhaps they had anticipated; but it surely will prove an incentive and stimulus to every member of the American Veterinary Medical Association to put forth renewed efforts in doing missionary work in this field of discord, strife, and personal animosities. The parent organization of our land has solved many trying problems in the past; spread its wings of peace over many discordant periods, and added strength and power and unity of purpose in many fields where these elements of strength were wanting. Such will be its mission in Michigan, and when it has achieved these much-to-be-desired results it may also hope that this field for future recruits may add to the schools that equip her graduates with strength and support to so qualify their graduates that they may become eligible as applicants for membership in the American Veterinary Medical Association.

